# P-987. It's Still Trichy: Impact of a Prescribing Alert on the Number of Women Receiving CDC-recommended Therapy for Trichomoniasis

**DOI:** 10.1093/ofid/ofaf695.1186

**Published:** 2026-01-11

**Authors:** Nicole Sunshine, Rachel M Kenney, Jacob Manteuffel, Nathan Everson, Christen J Arena, Erin Eriksson, Brian Church, Michael P Veve

**Affiliations:** University of South Carolina College of Pharmacy, MI; Henry Ford Hospital, Detroit, Michigan; Henry Ford Hospital, Detroit, Michigan; Henry Ford Hospital, Detroit, Michigan; University of Cincinnati, Cincinnati, OH; Henry Ford Jackson Hospital, Jackson, Michigan; Henry Ford Health, Detroit, Michigan; Eugene Applebaum College of Pharmacy and Health Sciences, Detroit, MI

## Abstract

**Background:**

The 2021 CDC STI treatment guidelines recommend a 7-day course of metronidazole or single-dose tinidazole for women with trichomoniasis due to improved patient outcomes compared to single-dose metronidazole therapy. The purpose of this study was to determine the impact of an electronic health record (EHR) alert on optimal trichomoniasis prescribing in women.

**Figure:**
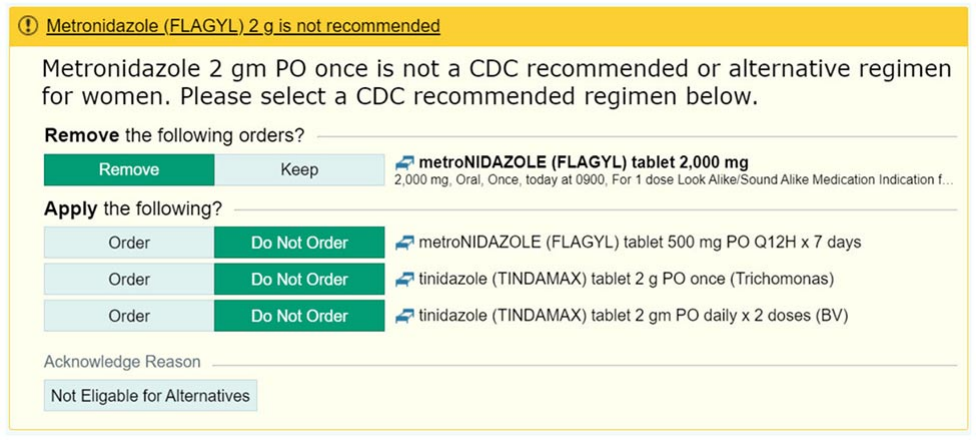

**Methods:**

This was an IRB-approved, single pre-test, post-test quasi-experiment of women >15 years positive for Trichomonas vaginalis who were treated in emergency department (ED) or outpatient clinic settings from 10/2023-12/2023 (pre-alert group) and 10/2024 to 12/2024 (post-alert group). An EHR alert was implemented 9/2024 that notifies prescribers that metronidazole 2g single-dose is not a recommended regimen and suggests CDC-recommended treatments (Figure 1). The primary outcome was the proportion of women who received a single dose of metronidazole 2g. Secondary outcomes were assessed in a cohort of patients from all care settings and included the number of clinical support alerts in the post-group and the proportion of women prescribed single-dose metronidazole after an alert.

**Results:**

285 patients were included: 49.8% pre- and 50.2% post-intervention. The median (IQR) patient age was 32 (IQR 23-41) years, 204 were black (71.6%), 65 were co-infected with bacterial vaginosis (22.8%), and 198 were symptomatic (69.5%). 8.5% of patients received single-dose metronidazole in the pre-alert group compared to 2.8% of patients in the post-alert group (unAdjOR, 0.312; 95%CI: 0.098-0.991). The clinical support alert fired 121 times across all care settings during the 3-month post-implementation period. 41% of these alerts were in ambulatory care settings; 50% in emergency department settings and 9% in inpatient labor and delivery units. There were 100 distinct patient orders that met inclusion criteria; 60 (50%) alerts were immediately accepted, and recommended therapy was chosen. Single-dose metronidazole treatment was ordered in 24 (20%) patients despite the alert.

**Conclusion:**

The implementation of a trichomoniasis clinical support tool was associated with a decrease in the prescription of single dose metronidazole for women treated for trichomoniasis.

**Disclosures:**

Jacob Manteuffel, MD, Cepheid: Honoraria|Gilead Sciences: Grant/Research Support|Indivior: Advisor/Consultant

